# Analytical Agreement of the GeneXpert HIV-1 Viral Load Assay Compared with the Abbott Alinity m HIV-1 Assay

**DOI:** 10.3390/diagnostics16172795

**Published:** 2026-08-31

**Authors:** Khulud A. Alhazmi, Sulaiman Bani AbdelRahman, Hala Altarawneh, Karem Ibrahem, Waiel S. Halabi, Abdulaziz Alsaedi, Turki Asiri, Bandar Hasan Saleh, Mohammed Mufrrih, Ahmad M. Alharbi, Khalid J. Shrwani, Radi Alsafi, Abdulelah Aljuaid, Mohannad A. Alzain, Mona Abdulrahman Alqarni, Nojoud Faqerah, Rawan Altalhi, Hatoon A. Niyazi, Noha A. Juma, Noura Daffa, Nariman Sindi, Wafaa Alhazmi, Ala A. Azhari, Basem A. Jawa

**Affiliations:** 1 Department of Microbiology and Parasitology, Faculty of Medicine, Umm Al-Qura University, Makkah 24382, Saudi Arabia; kahazemy@uqu.edu.sa; 2 Department of Microbiology and Pathology, Faculty of Medicine, Mutah University, P.O. Box 7, Mutah, Al-Karak 61710, Jordan; sulaiman.baniabdel-rahman@mutah.edu.jo (S.B.A.); hala.altarawneh@mutah.edu.jo (H.A.); 3 Department of Clinical Microbiology and Immunology, Faculty of Medicine, King Abdulaziz University, Jeddah 21589, Saudi Arabia; kaibrahem@kau.edu.sa (K.I.); absalsaede@kau.edu.sa (A.A.); bhsaleh@kau.edu.sa (B.H.S.); maaalqarni3@kau.edu.sa (M.A.A.); hniyazi@kau.edu.sa (H.A.N.); njmah@kau.edu.sa (N.A.J.); ndafaa@kau.edu.sa (N.D.); aaazhari@kau.edu.sa (A.A.A.); 4 Department of Clinical Microbiology Laboratory, King Abdulaziz University Hospital, Jeddah 21589, Saudi Arabia; 5 Department of Optometry, Faculty of Applied Medical Sciences, University of Jeddah, Jeddah 23218, Saudi Arabia; wshalabi@uj.edu.sa; 6 Department of Microbiology and Parasitology, Faculty of Medicine, King Abdulaziz University, Rabigh 21589, Saudi Arabia; talhomra@kau.edu.sa (T.A.); nfageerah@kau.edu.sa (N.F.); 7 Department of Medical Laboratory Sciences, Faculty of Applied Medical Sciences, King Abdulaziz University, Jeddah 21589, Saudi Arabia; mmufrrih@kau.edu.sa (M.M.); nsindi@kau.edu.sa (N.S.);; 8 Special Infectious Agents Unit BSL-3, King Fahd Medical Research Center, King Abdulaziz University, Jeddah 21589, Saudi Arabia; 9 Department of Clinical Laboratory Sciences, College of Applied Medical Sciences, Taif University, P.O. Box 11099, Taif 21944, Saudi Arabia; a.alharbii@tu.edu.sa (A.M.A.); ab.aljuaid@tu.edu.sa (A.A.); 10 Department of Medical Virology, Public Health Laboratory, Public Health Authority, P.O. Box 716, Jazan 45142, Saudi Arabia; kjshrwani@pha.gov.sa; 11 Department of Clinical Laboratory Sciences, Faculty of Applied Medical Sciences, Umm Al-Qura University, Makkah 24381, Saudi Arabia; rtsafi@uqu.edu.sa; 12 Department of Family Medicine, Faculty of Medicine, King Abdulaziz University, Jeddah 21589, Saudi Arabia; maaalzain1@kau.edu.sa; 13 Family Medicine and Chronic Diseases Research Unit, King Fahd Medical Research Center, King Abdulaziz University, Jeddah 21589, Saudi Arabia; 14 Department of Biological Sciences, College of Science, University of Jeddah, Jeddah 23445, Saudi Arabia; rmaltalhi@uj.edu.sa; 15 Vaccines and Immunotherapy Unit, King Fahd Medical Research Center, King Abdulaziz University, Jeddah 21589, Saudi Arabia; 16 Laboratory and Blood Bank, Alnoor Specialist Hospital, Makkah Health Cluster, Makkah 24241, Saudi Arabia

**Keywords:** HIV-1, PCR, GeneXpert, viral load, diagnostic accuracy, molecular diagnostics

## Abstract

**Background/Objectives:** Early and accurate quantification of Human Immunodeficiency Virus type 1 (HIV-1) viral load is essential for monitoring antiretroviral therapy and guiding clinical management. Although conventional real-time polymerase chain reaction (PCR) platforms provide reliable viral load measurement, they often require centralized laboratories and longer turnaround times. Cartridge-based systems such as the GeneXpert HIV-1 Viral Load assay offer a simplified testing workflow and may facilitate decentralized molecular testing. This study aimed to evaluate the qualitative and quantitative agreement between the GeneXpert HIV-1 Viral Load assay and the Abbott Alinity m HIV-1 assay. **Methods:** A total of 102 residual clinical Ethylenediaminetetraacetic acid (EDTA) plasma specimens were analyzed using both the GeneXpert HIV-1 Viral Load assay and the Abbott Alinity m HIV-1 assay. Qualitative agreement was evaluated using positive percent agreement (PPA), negative percent agreement (NPA), overall agreement, and Cohen’s κ coefficient. Quantitative agreement among specimens with paired quantifiable viral load results was assessed using Pearson’s correlation and Bland–Altman analyses. **Results:** Overall qualitative agreement between the two assays was 95.1%, with a PPA of 92.6%, an NPA of 100.0%, and a Cohen’s κ coefficient of 0.894, indicating almost perfect agreement. Among the 57 specimens with paired quantifiable viral load results, a very strong positive correlation was observed (Pearson’s r = 0.9972; 95% CI: 0.9952–0.9984; *p* < 0.001). Bland–Altman analysis demonstrated a minimal mean bias of −0.018 log_10_ IU/mL, with 95% limits of agreement ranging from −0.175 to 0.139 log_10_ International Units per milliliter (IU/mL). All paired quantitative differences were within the clinically accepted agreement threshold of ±0.5 log_10_ IU/mL. **Conclusions:** The GeneXpert HIV-1 Viral Load assay demonstrated excellent qualitative and quantitative agreement with the Abbott Alinity m HIV-1 assay. These findings support the potential use of the GeneXpert HIV-1 Viral Load assay as an alternative testing platform for routine HIV-1 viral load monitoring in appropriate clinical settings.

## 1. Introduction

Significant advancements have occurred in the areas of prevention, diagnosis, and treatment for HIV infection, yet it remains a public health challenge [1]. To prevent further transmission of HIV to other individuals, delay disease progression, and begin antiretroviral therapy (ART) on time, a timely and accurate diagnosis of HIV is critical [2]. Developing rapid and accurate strategies for diagnosing HIV is imperative for achieving global targets for controlling the HIV epidemic [3]. The ongoing global health strategic plan supports this goal as well.

PCR-base detection methods are now regarded as a vital element in the diagnosis of HIV infection, and in particular, for the identification of HIV in infants/newborns and for resolving inconclusive serologic test results [4]. Real-time PCR tests provide a means for the direct identification of HIV-1 nucleic acid, thus providing high analytical sensitivity and specificity [5]. Traditional laboratory-based Real-Time PCR platforms are usually employed as reference methods; however, they often require elaborate infrastructure, highly trained personnel and long turnaround times due to batch testing and centralized testing platforms [5].

Accurate molecular detection of HIV is important for the diagnostic and therapeutic management of persons infected with HIV [6]. PCR-based techniques allow for the direct quantification and identification of HIV-1 nucleic acid and provide valuable information for the initiation of antiretroviral therapy (ART), especially in early infections or populations in whom serological testing may be less reliable [7]. Early acquisition of a molecular confirmation provides an opportunity for prompt treatment initiation, leading to a decrease in both mortality and morbidity from HIV infection, as well as a decrease in the transmission of the virus [7].

Several literature reviews confirm the importance of using quantitative HIV PCR testing when evaluating the effectiveness of ART in responding to therapy [7]. Monitoring the HIV viral load is the primary measure of therapeutic efficacy and is instrumental in assessing whether virus suppression has occurred, treatment compliance has been achieved, and/or treatment regimens need to be changed [8]. Viral suppression has clinically significant benefits and has caused dramatic improvement of the immune system in the form of a measurable increase in the immune system, as measured by highly sensitive PCR tests, in addition to causing a lower rate of developing an opportunistic infection. In addition, achieving long-term suppression of a patient’s viral load has a beneficial effect on the long-term outcomes of being treated for HIV [9]. The clinical decision-making surrounding HIV treatment is directly impacted by the reliability and analytical sensitivity of the PCR assay [7].

Therefore, the PCR assay is required for both low viremia detection and early detection of virological failure (VF) while on ART [10]. Molecular tests used to identify viral rebound are probably more effective at identifying these rebounds prior to clinical or immunologic deterioration, allowing for timely intervention (i.e., treatment intensification or adherence counseling) [11]. Variations in the assay sensitivity and detection threshold of PCR tests could potentially influence the diagnosis of low-level viremia. Thus, it is critical that the clinical relevance of PCR tests be established for the purpose of monitoring treatment [12].

The introduction of rapid, cartridge-based PCR platforms has dramatically enhanced the management of HIV therapy by reducing turnaround time and enabling near point-of-care VL testing [13]. Immediate PCR results enable same-day or prompt treatment decisions, promote retention in care and allow rapid adjustments to ART [14]. The current turnaround times for reporting results via the central laboratory testing process, as well as the general inefficiencies associated with the current centralized laboratory testing process, can lead to delays in diagnosis and result in a significant delay in the delivery of appropriate antiretroviral therapy to HIV-positive patients [15].

The GeneXpert platform (Cepheid) is a cartridge-based real-time PCR technology that has recently become widely used in clinical laboratories [16]. The GeneXpert real-time PCR methodology is designed to automate the sample processing, amplification, and detection phases of testing through a closed system with limited manual processes, thus minimizing the risk of contamination [17]. In addition, the GeneXpert platform offers rapid turnaround times and is easy to use, thus making it an attractive option for clinical laboratories compared to conventional real-time open PCR methodologies [18]. Yet, assay design variation and analytical performance need to be critically considered against established real-time PCR assays before the use of the GeneXpert platform for routine HIV-1 viral load testing [19].

Various studies have already assessed the performance of the GeneXpert HIV-1 viral load assay against other well-established molecular assays, but the performance of the assay may differ depending on the comparator platform, the characteristics of the specimens, the viral load distribution and the laboratory environment. Laboratories looking to use more than one test system for rapid, random-access testing and high-throughput automated molecular testing will find it important to have evidence comparing the Abbott Alinity m system with the GeneXpert HIV-1 assay. In addition, the agreement in the clinically relevant range of viral loads, especially in the proximity of the lower limits of quantification (LLoQ), must be carefully considered, as small inter-assay differences could influence the interpretation of low-level viremia and virological monitoring.

Although several studies have evaluated the analytical performance of the GeneXpert HIV-1 Viral Load assay in comparison with established laboratory-based platforms, published data from Saudi Arabia remain limited. To our knowledge, published data evaluating the analytical agreement between the GeneXpert HIV-1 Viral Load assay and the Abbott Alinity m HIV-1 assay in Saudi Arabia are limited. Therefore, this study aimed to evaluate the qualitative and quantitative agreement between the GeneXpert HIV-1 Viral Load assay and the Abbott Alinity m HIV-1 assay using residual clinical plasma specimens from a Saudi clinical laboratory.

## 2. Materials and Methods

### 2.1. Study Design and Setting

A comparative analysis was performed to evaluate the analytical performance and agreement of two PCR-based molecular platforms for HIV-1 testing. The performance of the GeneXpert HIV-1 PCR assay (Cepheid, Sunnyvale, California, United State of America (USA)) was compared with that of the Abbott Alinity m molecular system (Abbott Diagnostics, Abbott Park, Illinois, USA), which served as the comparator assay in this study. Abbott Alinity m was selected as the comparator because it is an established, fully automated, high-throughput real-time PCR platform used for routine HIV-1 viral load testing in the study laboratory, thereby allowing the performance of the cartridge-based GeneXpert system to be evaluated against the laboratory’s existing routine molecular testing platform. The study was conducted in a clinical diagnostic laboratory routinely providing HIV molecular testing services.

### 2.2. Study Samples

A total of 102 de-identified residual EDTA plasma specimens were included in this study. All specimens were obtained from patients with previously diagnosed HIV-1 infection who attended an infectious diseases clinic for routine HIV-1 viral load monitoring. Whole blood was collected in 8 mL EDTA tubes, and plasma was separated according to routine laboratory procedures. Following completion of routine diagnostic testing, residual plasma specimens with sufficient remaining volume were used for the comparative analysis. Because the specimens were de-identified residual clinical samples, individual HIV serology results were not available to the investigators; however, all specimens originated from patients with previously diagnosed HIV-1 infection undergoing routine viral load monitoring. Specimens with inadequate residual volume or evidence of pre-analytical compromise were excluded.

Specimens were selected based on having adequate residual plasma volume and were purposively chosen to span a wide spectrum of HIV-1 viral load, ranging from undetectable to low, intermediate, and high concentrations. This sampling approach was chosen to allow evaluation of agreement between the two molecular platforms across their analytical ranges. All specimens were de-identified prior to inclusion in the study, and individual demographic or clinical information, including age, sex, ART status, treatment history, and duration of HIV infection, was not available for analysis.

For the qualitative agreement analysis, all 102 specimens were analyzed on both platforms. Based on the Abbott Alinity m comparator results, 68 specimens had detectable HIV-1 Ribonucleic acid (RNA) and 34 had undetectable HIV-1 RNA. Thus, the 68 detected specimens were not selectively sampled for the analysis; they represented the subset of the full 102-specimen cohort that was detected by the comparator assay.

Because of the study period, no a priori statistical power calculation was performed, and the sample size was based on the number of eligible residual clinical specimens with sufficient available plasma volume. A total of 102 specimens were therefore included in the comparison.

Given the exploratory method-comparison design and the availability-based purposive sampling approach, the study was intended to provide preliminary evidence of agreement between the two molecular platforms rather than population-level estimates of diagnostic performance. The absence of an a priori sample size calculation and the relatively limited sample size are acknowledged as limitations of the study.

### 2.3. Sample Storage and Handling

After preliminary routine testing, all specimens were aliquoted to facilitate comparative analysis on both platforms. Samples were stored at −80 °C for up to three months before testing. Frozen specimens were thawed once and processed immediately according to the manufacturers’ instructions to preserve nucleic acid integrity and minimize degradation. Specimens yielding invalid or error results on either assay were retested according to the manufacturer’s instructions until a valid result was obtained. The final valid results were used for all qualitative and quantitative agreement analyses. Repeat testing was not performed solely to resolve discordant results between the two assays.

### 2.4. GeneXpert HIV-1 Viral Load Assay

HIV-1 viral load testing with the GeneXpert HIV-1 Viral Load assay (Cepheid, USA) was performed according to the manufacturer’s instructions. The GeneXpert platform is an automated, cartridge-based real-time PCR system that integrates nucleic acid extraction, amplification, and detection within a closed system. Results were reported as detected or not detected, with quantitative HIV-1 RNA values provided when within the assay’s quantifiable range. According to the manufacturer’s Instructions for Use, the assay has a limit of detection (LoD) of ≤21.1 copies/mL and a LLoQ of 40 copies/mL. Results below the LLoQ of 40 copies/mL were interpreted as below the quantifiable range and were reported as “<40 copies/mL” rather than assigned a numerical viral-load value.

### 2.5. Abbott Alinity m HIV-1 Assay

The Abbott Alinity m HIV-1 assay (Abbott Molecular) was used as the comparator assay, and testing was performed according to the manufacturer’s instructions. The platform uses automated nucleic acid extraction and real-time PCR amplification for the detection and quantification of HIV-1 RNA. Abbott Alinity m was selected as the comparator because it was the established high-throughput molecular platform used for routine HIV-1 viral load testing in the study laboratory. According to the manufacturer’s Instructions for Use, the assay has a limit of detection of 20 copies/mL and a lower limit of quantification of 20 copies/mL.

However, Abbott Alinity m is a commercial molecular assay rather than an independent gold-standard method, and its results may be influenced by assay-specific analytical characteristics, including its limits of detection and quantification, target regions, calibration, and measurement variability. Therefore, the findings of the present study were interpreted as an assessment of agreement between two molecular platforms rather than validation of GeneXpert against an absolute gold standard.

The analytical characteristics of the GeneXpert HIV-1 Viral Load assay and the Abbott Alinity m HIV-1 assay, as reported in the respective manufacturers’ Instructions for Use, are summarized in Table 1.

### 2.6. Analytical Characteristics of the Assays

The analytical characteristics of the two assays, including specimen type, LoD, LLoQ, and reportable range, were obtained from the respective manufacturers’ Instructions for Use. The GeneXpert HIV-1 Viral Load assay has a LoD of ≤21.1 copies/mL and an LLoQ of 40 copies/mL, whereas the Abbott Alinity m HIV-1 assay has a LoD and LLoQ of 20 copies/mL. The corresponding reportable ranges are 40–10,000,000 copies/mL for GeneXpert and 20–10,000,000 copies/mL for Abbott Alinity m.

Viral load results were retained in IU/mL because this was the reporting unit configured on both assay platforms in the study laboratory. The manufacturers provide analytical performance characteristics in copies/mL; therefore, the assay LoD and LLoQ are presented in copies/mL as specified in the respective Instructions for Use.

### 2.7. Comparative Performance Evaluation

Qualitative agreement in HIV-1 RNA detection between the GeneXpert HIV-1 PCR assay and the Abbott Alinity m comparator assay was evaluated using a 2 × 2 contingency table. Because Abbott Alinity m was used as a commercial comparator assay rather than an independent gold standard, PPA, NPA, positive predictive value (PPV), negative predictive value (NPV), overall agreement, and Cohen’s kappa (κ) coefficient were calculated together with their corresponding 95% confidence intervals. Quantitative agreement between the two assays among specimens with paired quantifiable viral load results was evaluated using Pearson’s correlation analysis and Bland–Altman analysis. Pearson’s correlation coefficient with its corresponding 95% confidence interval was calculated to assess the strength of the linear association between the two assays. Bland–Altman analysis was used as the primary method for assessing agreement, and the mean bias, 95% limits of agreement, and their corresponding 95% confidence intervals were calculated and reported.

### 2.8. Statistical Analysis

Statistical analyses were performed using GraphPad Prism version 10 (GraphPad Software, San Diego, CA, USA) and IBM SPSS Statistics for Windows, Version 26.0 (IBM Corp., Armonk, NY, USA). Continuous variables were summarized using appropriate descriptive statistics, and categorical variables were summarized as frequencies and percentages. Qualitative and quantitative agreement analyses were performed as described in Section 2.6. Pearson’s correlation analysis was applied to the log_10_-transformed paired viral load measurements to assess the strength of the linear association between the two assays. Bland–Altman analysis was used to evaluate agreement between the assays by estimating the mean bias and the 95% limits of agreement. Ninety-five percent confidence intervals were calculated where appropriate. A two-sided *p*-value of <0.05 was considered statistically significant.

### 2.9. Ethical Considerations

The Biomedical Research Ethics Committee at Umm Al-Qura University reviewed and approved this study (Approval No. HAPO-02-K-012-2025-01-2501). The study was conducted according to the ethical requirements of the testing facility, and all identifiers of subjects were removed prior to analysis to ensure confidentiality. The samples were processed during routine diagnostic testing.

## 3. Results

### 3.1. Qualitative Agreement Between GeneXpert and Abbott Alinity m

A total of 102 specimens obtained from patients with previously confirmed HIV infection were included in the qualitative agreement analysis. Of these, 68 had detectable HIV-1 RNA according to the Abbott Alinity m comparator assay, whereas 34 had undetectable HIV-1 RNA on both platforms. Among the 68 specimens with HIV-1 RNA detected by Abbott Alinity m, 63 were also detected by GeneXpert, while five low-level specimens were reported as undetectable by GeneXpert. No specimens were detected by GeneXpert while being undetectable by Abbott Alinity m (Table 2).

Overall, concordant qualitative results were observed in 97 of 102 specimens (95.1%). The PPA was 92.6%, and the NPA was 100.0%. The PPV and NPV were 100.0% and 87.2%, respectively. Cohen’s κ was 0.894, indicating almost perfect agreement between the two assays (Table 3).

### 3.2. Quantitative Agreement Between GeneXpert and Abbott Alinity m

Of the 102 specimens included in the study, 57 specimens had paired quantifiable HIV-1 viral load results suitable for quantitative comparison between the two platforms. The remaining specimens had either undetectable HIV-1 RNA or results below the quantifiable range of one or both assays and were therefore excluded from analyses requiring paired numerical viral load measurements (Table 4).

**Figure 1:**
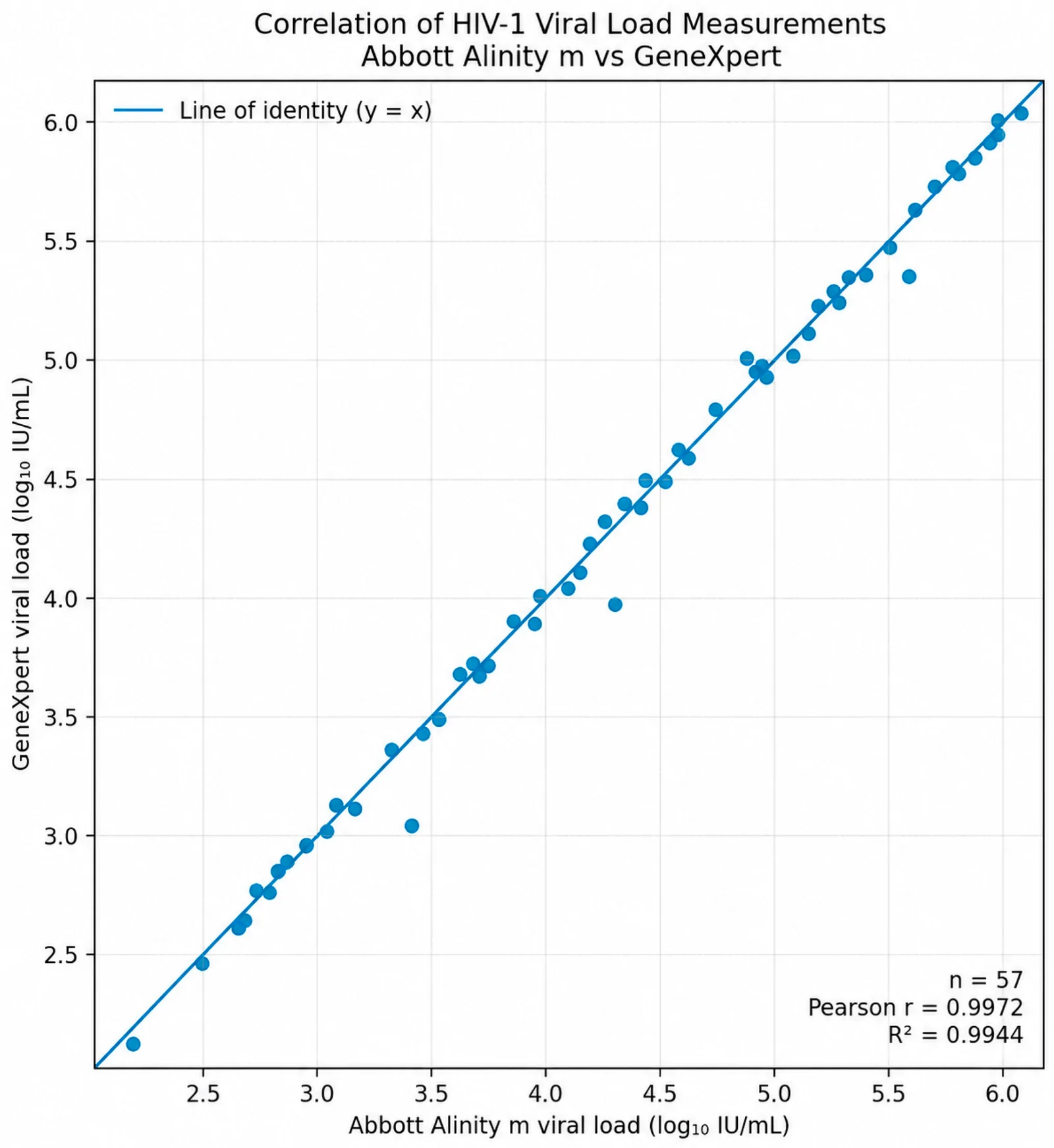
Correlation between paired quantifiable HIV-1 viral load measurements obtained using the Abbott Alinity m and GeneXpert HIV-1 Viral Load assays. Each point represents one specimen with quantifiable results on both platforms (n = 57). Viral load measurements are expressed as log_10_ IU/mL. The diagonal line represents the line of identity (y = x). Pearson’s correlation coefficient was r = 0.9972 (R^2^ = 0.9944).

A strong positive linear correlation was observed between the log_10_-transformed viral load measurements obtained using Abbott Alinity m and GeneXpert (Pearson’s r = 0.9972; 95% CI: 0.9952–0.9984; *p* < 0.001), indicating a very strong linear association between the quantitative measurements generated by the two assays (Figure 1). However, because correlation measures association rather than agreement, agreement between the two assays was primarily assessed using Bland–Altman analysis.

**Figure 2:**
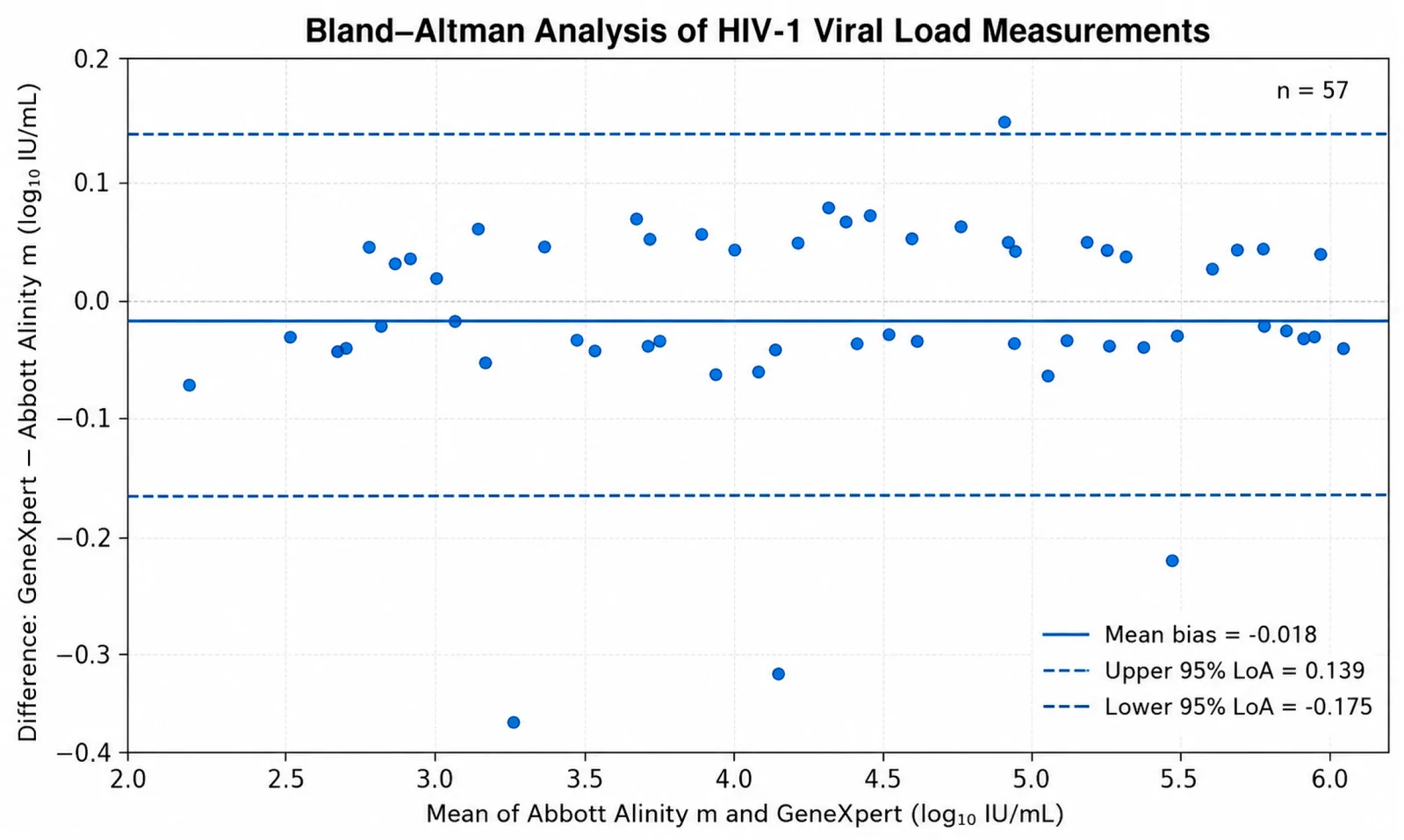
Bland–Altman analysis of agreement between Abbott Alinity m and GeneXpert HIV-1 viral load measurements. The plot illustrates the differences between paired log_10_ transformed HIV-1 viral load measurements obtained using the GeneXpert HIV-1 Viral Load assay and the Abbott Alinity m assay (GeneXpert − Abbott Alinity m) against their mean values (n = 57). The solid horizontal line represents the mean bias (−0.018 log_10_ IU/mL), while the dashed lines represent the 95% limits of agreement (−0.175 to 0.139 log_10_ IU/mL). All paired quantitative differences were within the predefined clinically accepted agreement threshold of ±0.5 log_10_ IU/mL.

Using GeneXpert minus Abbott Alinity m to define the paired difference, Bland–Altman analysis demonstrated a mean bias of −0.018 log_10_ IU/mL, with a standard deviation of 0.080 log_10_ IU/mL. The 95% limits of agreement ranged from −0.175 to 0.139 log_10_ IU/mL. All observed paired quantitative differences were within the clinically accepted agreement threshold of ±0.5 log_10_ IU/mL (Figure 2). These findings demonstrate excellent quantitative agreement between the GeneXpert HIV-1 Viral Load assay and the Abbott Alinity m assay across the evaluated viral load range.

## 4. Discussion

The comparative study showed good analytical agreement between the GeneXpert HIV-1 Viral Load assay and the Abbott Alinity m HIV-1 assay over a wide dynamic range. Quantitative agreement was excellent, with an excellent Pearson correlation coefficient (r = 0.9972), a small mean bias (−0.018 log_10_ IU/mL), and narrow 95% limits of agreement (−0.175 to 0.139 log_10_ IU/mL). Both assays produced highly comparable viral load measurements over five orders of magnitude. The differences between all paired measurements were smaller than the clinically accepted threshold of ±0.5 log_10_ IU/mL, indicating that these differences are unlikely to influence clinical decision-making. No clinically relevant systematic bias was observed despite intrinsic technological differences between the two platforms, including nucleic acid extraction methods and amplification chemistry.

This evaluation is consistent with the results of earlier studies with the GeneXpert HIV-1 Viral Load assay. A systematic review and meta-analysis of 13 studies showed that the assay had good analytical performance, with a pooled Pearson correlation coefficient of around 0.94 compared with the gold standard laboratory-based viral load assay, and a pooled Bland–Altman limit of agreement generally within 0.35 log_10_ copies/mL [20]. Likewise, the GeneXpert assay was shown to have a Pearson correlation coefficient of 0.985 and 97.7% of quantified specimens within ±0.5 log_10_ copies/mL in Bland–Altman analysis in the multicenter clinical evaluation that compared the two assays (Abbott RealTime HIV-1 assay) [21]. Very recently, the Abbott Xpert HIV-1 Viral Load XC assay was also evaluated against the Abbott Alinity m and Abbott RealTime assays, with excellent results–including low mean bias and good correlation over a wide viral load range–demonstrating the robustness of the GeneXpert platform [22]. These reports are consistent with our findings, which demonstrated a Pearson correlation coefficient of 0.9972, a very small mean bias of −0.018 log_10_ IU/mL, and all paired measurements within the clinically accepted agreement threshold of ±0.5 log_10_ IU/mL. In addition, the high positive percent agreement (92.6%), negative percent agreement (100%), overall agreement (95.1%), and Cohen’s κ coefficient (0.894) further demonstrate the excellent qualitative agreement between the two assays for HIV-1 RNA detection. Minor differences among studies are likely attributable to variations in comparator assays, study populations, HIV-1 subtype distribution, specimen selection, laboratory workflows, and statistical approaches. Collectively, these findings support the reliability and clinical applicability of the GeneXpert HIV-1 Viral Load assay for routine HIV viral load monitoring in both centralized and decentralized laboratory settings [23].

Based on their intended design and previously published experience, both platforms offer distinct operational characteristics that may influence their use in different laboratory settings. Cartridge-based systems such as the GeneXpert HIV-1 Viral Load assay are generally suited to low-volume or urgent testing because they enable random-access analysis without requiring batch processing. In contrast, high-throughput automated platforms such as the Abbott Alinity m system are designed to process larger numbers of specimens efficiently in laboratories with high testing volumes. These operational characteristics were not quantitatively evaluated in the present study but may be important considerations when selecting molecular testing platforms for different clinical and laboratory environments.

The excellent analytical agreement demonstrated in the present study suggests that the two platforms may serve complementary roles in routine HIV-1 viral load testing. High-throughput automated systems remain well suited for centralized laboratories processing large specimen volumes, whereas cartridge-based platforms may provide greater flexibility for decentralized laboratories or situations requiring rapid access to viral load results.

The excellent analytical agreement observed between the two assays suggests that the GeneXpert HIV-1 Viral Load assay may represent a suitable alternative testing platform for routine HIV-1 viral load monitoring in appropriate clinical settings.

The observed mean bias of −0.018 log_10_ IU/mL is substantially smaller than the magnitude of change generally considered clinically meaningful in HIV viral load monitoring and is unlikely to influence clinical decision-making. In particular, the bias is unlikely to affect patient classification around clinically relevant viral load thresholds, including 200 copies/mL, which is used in some clinical settings to assess virological suppression, and the WHO-recommended threshold of 1000 copies/mL for defining virological failure and the need for enhanced adherence support or treatment evaluation. Furthermore, all paired measurements were within the clinically accepted agreement threshold of ±0.5 log_10_ IU/mL, suggesting that the GeneXpert HIV-1 Viral Load assay provides results that are clinically comparable to those obtained with the Abbott Alinity m assay for routine patient monitoring.

To our knowledge, published data evaluating the analytical agreement between the GeneXpert HIV-1 Viral Load assay and the Abbott Alinity m HIV-1 assay in Saudi Arabia are limited. Therefore, our findings provide local method-comparison data supporting the performance of the GeneXpert platform in a Saudi clinical laboratory and contribute evidence for its potential implementation in routine HIV-1 viral load testing.

This study has several limitations. First, it was conducted at a single center, which may limit the generalizability of the findings to other healthcare settings and populations. Second, the study included a relatively small sample of 102 residual clinical specimens, and the sample size was determined by specimen availability rather than an a priori statistical power calculation. Consequently, the study may have had limited statistical power to detect small differences between the two assays, and the precision of the estimated performance measures may be limited. Third, specimens were selected non-consecutively based on the availability of sufficient residual plasma volume and were purposively chosen to represent a broad range of HIV-1 viral loads. This sampling approach may have introduced selection bias, and the viral load distribution in the study sample may not reflect that observed in the routine clinical population. Fourth, because de-identified residual clinical specimens were used, individual-level demographic and clinical information, including age, sex, antiretroviral therapy status, treatment history, and duration of HIV infection, was unavailable, limiting the assessment of assay performance across specific patient subgroups. In addition, HIV-1 subtype analysis was not performed; therefore, the performance of the assays for the different viral subtypes could not be assessed. In addition, reproducibility testing was not performed across different operators/instrumentations and evaluation of inter-operator/inter-instrument variability was limited. Furthermore, this study has been mainly analytical, comparing the two molecular platforms. There was no prospective collection of operational performance metrics such as turnaround time, hands-on time, throughput, invalid/error rates and retesting frequency during routine lab testing, which prevented quantitative comparison between the two platforms. In addition, a formal cost-effectiveness analysis was beyond the scope of the present study. These findings should be confirmed by larger, adequately powered multicenter studies over consecutive or systematically selected specimens and with diverse HIV-1 subtypes to assess assay performance in various clinical populations.

## 5. Conclusions

This study showed good qualitative and quantitative correlation between the GeneXpert HIV-1 Viral Load assay and Abbott Alinity m HIV-1 assay for HIV-1 viral load testing. Agreement was high across the viral load measured range, and the limits of agreement were very narrow, with a small mean bias, suggesting that the GeneXpert HIV-1 Viral Load assay is comparable to the Abbott Alinity m assay. These findings support the potential use of the GeneXpert HIV-1 Viral Load assay as an alternative testing platform for routine HIV-1 viral load monitoring in appropriate clinical settings. However, because this was a single-center study using a limited number of purposively selected residual clinical specimens, larger multicenter studies including diverse HIV-1 subtypes and prospective evaluation of operational performance and cost-effectiveness are warranted to confirm these findings.

## Figures and Tables

**Table 1:** Analytical characteristics of the GeneXpert HIV-1 Viral Load assay and the Abbott Alinity m HIV-1 assay according to the manufacturers’ Instructions for Use.

Characteristic	GeneXpert HIV-1 Viral Load (Cepheid)	Abbott Alinity m HIV-1 (Abbott Molecular)
Assay principle	Automated cartridge-based real-time reverse transcription polymerase chain reaction (RT-PCR)	Automated real-time RT-PCR
Target analyte	HIV-1 RNA	HIV-1 RNA
Specimen type	EDTA plasma	EDTA plasma (quantitative monitoring); plasma and serum (supplemental confirmation)
Limit of detection (LoD)	21.1 copies/mL (95% CI: 16.1–26.0; maximum observed Probit estimate using the World Health Organization (WHO) 3rd International Standard)	20 copies/mL
Lower limit of quantification (LLoQ)	40 copies/mL (1.60 log_10_ copies/mL)	20 copies/mL (1.30 log_10_ copies/mL)
Linear/reportable (dynamic) range	40–10,000,000 copies/mL (1.60–7.00 log_10_ copies/mL)	20–10,000,000 copies/mL (1.30–7.00 log_10_ copies/mL)
Precision (manufacturer claim)	Precision and reproducibility established according to The Clinical and Laboratory Standards Institute (CLSI) guidelines across multiple sites, operators, reagent lots, and instruments; acceptable trueness and precision demonstrated at the LLoQ.	Precision established according to the manufacturer’s validation studies and described in the package insert for the reportable range
Automation	Fully automated closed-cartridge system integrating extraction, amplification, detection, and quantification	Fully automated integrated platform for nucleic acid extraction, amplification, detection, and quantification
Intended use	Quantification of HIV-1 RNA in plasma for monitoring HIV-1 infection	Detection and quantification of HIV-1 RNA for monitoring HIV-1 infection and supplemental confirmation of HIV-1 infection

Cepheid. Xpert^®^ HIV-1 Viral Load Instructions for Use (IFU), Revision N. (https://infomine.cepheid.com/sites/default/files/2023-08/Xpert%20HIV-1%20Viral%20Load%20ENGLISH%20IFU%20301-3068%2C%20Rev.%20N.pdf?utm_source=chatgpt.com). (accessed on 1 July 2026). Abbott Molecular. Alinity m HIV-1 Package Insert/Instructions for Use (FDA-cleared IFU) (https://www.fda.gov/vaccines-blood-biologics/alinity-m-hiv-1?utm_source=chatgpt.com). (accessed on 4 July 2026).

**Table 2:** Qualitative Agreement in HIV-1 RNA Detection Between GeneXpert and Abbott Alinity m (Number (n) = 102).

GeneXpert Result	Abbott Alinity m: Detected	Abbott Alinity m: Not Detected	Total
Detected	63	0	63
Not detected	5	34	39
Total	68	34	102

Note: This table means: GeneXpert Detected + Abbott Detected = 63, GeneXpert Detected + Abbott Not detected = 0, GeneXpert Not detected + Abbott Detected = 5, GeneXpert Not detected + Abbott Not detected = 34.

**Table 3:** Qualitative agreement measures between the GeneXpert HIV-1 PCR assay and Abbott Alinity m (n = 102).

Agreement Measure	Estimate	95% CI
Positive percent agreement (PPA)	92.6%	83.7–97.6%
Negative percent agreement (NPA)	100.0%	89.7–100.0%
Positive predictive value (PPV)	100.0%	94.3–100.0%
Negative predictive value (NPV)	87.2%	72.6–95.7%
Overall agreement	95.1%	88.9–98.4%
Cohen’s κ	0.894	0.803–0.984

Note: Abbott Alinity m was used as the comparator assay. CI, confidence interval; PPA, positive percent agreement; NPA, negative percent agreement; PPV, positive predictive value; NPV, negative predictive value. Exact Clopper–Pearson 95% confidence intervals were calculated for PPA, NPA, PPV, NPV, and overall agreement. The 95% confidence interval for Cohen’s κ was calculated using the asymptotic standard error.

**Table 4:** Paired quantitative HIV-1 viral load measurements obtained using Abbott Alinity m and GeneXpert (n = 57).

Sample	Abbott (IU/mL)	Abbott (log_10_ IU/mL)	GeneXpert (IU/mL)	GeneXpert (log_10_ IU/mL)	Difference (GeneXpert − Abbott), log_10_ IU/mL
1	156	2.193	134	2.127	−0.066
2	2574	3.411	1110	3.045	−0.365
3	19,940	4.300	9450	3.975	−0.324
4	27,167	4.434	31,400	4.497	0.063
5	385,603	5.586	226,000	5.354	−0.232
6	450	2.653	410	2.613	−0.040
7	1200	3.079	1350	3.130	0.051
8	8900	3.949	7800	3.892	−0.057
9	55,000	4.740	62,000	4.792	0.052
10	120,000	5.079	105,000	5.021	−0.058
11	950,000	5.978	890,000	5.949	−0.028
12	310	2.491	290	2.462	−0.029
13	4200	3.623	4800	3.681	0.058
14	12,500	4.097	11,000	4.041	−0.056
15	88,000	4.944	95,000	4.978	0.033
16	250,000	5.398	230,000	5.362	−0.036
17	670	2.826	710	2.851	0.025
18	3400	3.531	3100	3.491	−0.040
19	18,000	4.255	21,000	4.322	0.067
20	75,000	4.875	102,000	5.009	0.134
21	500,000	5.699	540,000	5.732	0.033
22	1,200,000	6.079	1,100,000	6.041	−0.038
23	890	2.949	920	2.964	0.014
24	5600	3.748	5200	3.716	−0.032
25	22,000	4.342	25,000	4.398	0.056
26	140,000	5.146	130,000	5.114	−0.032
27	600,000	5.778	650,000	5.813	0.035
28	1100	3.041	1050	3.021	−0.020
29	9400	3.973	10,200	4.009	0.035
30	33,000	4.519	31,000	4.491	−0.027
31	180,000	5.255	195,000	5.290	0.035
32	750,000	5.875	710,000	5.851	−0.024
33	540	2.732	590	2.771	0.039
34	2900	3.462	2700	3.431	−0.031
35	15,500	4.190	17,000	4.230	0.040
36	92,000	4.964	85,000	4.929	−0.034
37	410,000	5.613	430,000	5.633	0.021
38	1450	3.161	1300	3.114	−0.047
39	7200	3.857	8000	3.903	0.046
40	42,000	4.623	39,000	4.591	−0.032
41	210,000	5.322	225,000	5.352	0.030
42	880,000	5.944	820,000	5.914	−0.031
43	610	2.785	580	2.763	−0.022
44	4800	3.681	5300	3.724	0.043
45	26,000	4.415	24,000	4.380	−0.035
46	155,000	5.190	170,000	5.230	0.040
47	640,000	5.806	610,000	5.785	−0.021
48	730	2.863	780	2.892	0.029
49	5100	3.708	4700	3.672	−0.035
50	38,000	4.580	42,000	4.623	0.044
51	190,000	5.279	175,000	5.243	−0.036
52	950,000	5.978	1,020,000	6.009	0.031
53	480	2.681	440	2.643	−0.038
54	2100	3.322	2300	3.362	0.040
55	14,000	4.146	12,800	4.107	−0.039
56	82,000	4.914	90,000	4.954	0.041
57	320,000	5.505	300,000	5.477	−0.028

Note: HIV-1 viral load results with paired and quantifiable results were measured on both platforms and included in the quantitative comparison. Viral load measurements are presented in both IU/mL and log_10_ IU/mL. Log_10_ transformations were carried out for statistical analysis. The differences were calculated as GeneXpert–Abbott Alinity m (log_10_ IU/mL). Results from samples where either assay did not detect the presence of the virus or the concentration was too low to be quantified were not included in this quantitative comparison.

## Data Availability

The original contributions presented in this study are included in the article. Further inquiries can be directed to the corresponding author.

## Abbreviations

The following abbreviations are used in this manuscript:

HIV-1	Human Immunodeficiency Virus type 1
PCR	Polymerase chain reaction
EDTA	Ethylenediaminetetraacetic acid
PPA	positive percent agreement
NPA	negative percent agreement
VF	Virological failure
LLoQ	Lower limits of quantification
RNA	Ribonucleic acid
LoD	Llimit of detection
RT-PCR	Reverse transcription polymerase chain reaction
CLSI	Clinical and Laboratory Standards Institute
IFU	Instructions for Use
PPV	Positive predictive value
NPV	Negative predictive value
IU/mL	International Units per milliliter
κ	kappa
n	Number
USA	United State of America
WHO	World Health Organization
